# Enhancement of SARS-CoV-2 vaccine-induced immunity by a Toll-like receptor 7 agonist adjuvant

**DOI:** 10.1038/s41392-023-01485-6

**Published:** 2023-05-24

**Authors:** Gen Li, Meixing Yu, Qiong Ke, Jing Sun, Yanwen Peng, Chuanfeng Xiong, Olivia Monteiro, COVID-19 Infection and Immunity Investigation Group, Jincun Zhao, Andy P. Xiang

**Affiliations:** 1 Guangzhou Women and Children’s Medical Center, Guangzhou Medical University, Guangzhou, China; 2 Center for Stem Cell Biology and Tissue Engineering, Key Laboratory for Stem Cells and Tissue Engineering, Ministry of Education, Sun Yat-sen University, Guangzhou, China; 3 State Key Laboratory of Respiratory Disease, National Clinical Research Center for Respiratory Disease, Guangzhou Institute of Respiratory Health, First Affiliated Hospital, Guangzhou Medical University, Guangzhou, China; 4 Center for Biomedicine and Innovations, Faculty of Medicine, Macau University of Science and Technology, Macau, China

**Keywords:** Vaccines, Infectious diseases

**Dear Editor**,

Severe acute respiratory syndrome coronavirus 2 (SARS-CoV-2) is an enveloped positive-sense single-stranded RNA (ssRNA) virus responsible for the global COVID-19 pandemic. Although vaccines for SARS-CoV-2 have been developed and applied worldwide, research on vaccine adjuvant is lacking. Viral infections normally elicit a range of innate immune responses through the activation of pattern recognition receptors (PRRs), which have evolved to recognize a range of pathogen-associated molecular patterns (PAMPs). These innate immune responses, in turn, lead to the production of cytokines that boost adaptive immunity, which confer long-term protection against infection. The use of adjuvants to activate PRRs and boost immune responses during vaccination is well established.^1^ TLR7 recognizes ssRNA within endosomes and has been implicated in anti-viral immune responses. Adjuvants that activate TLR7 have previously been shown to boost protective immunity when administrated with the hepatitis C virus (an ssRNA virus) vaccine.^2^ Imiquimod, a TLR7 agonist, is an FDA-approved topical treatment for genital warts, superficial basal cell carcinoma, and actinic keratosis. We explored whether the addition of imiquimod as an adjuvant to a recombinant vaccine comprising the SARS-CoV-2 spike glycoprotein RBD^3^ could enhance anti-SARS-CoV-2 immunity.

Cynomolgus macaques (*Macaca fascicularis*) were initially immunized by three subcutaneous injections with the RBD vaccine in the presence of topical imiquimod (100 mg) over the site of injection on days 0, 7, and 14. Sera were collected from the NHPs at days 0, 14, 28, and 84 and examined for their SARS-CoV-2 neutralizing activity by pseudovirus assay. The addition of TLR7 agonist to the RBD vaccine (RBD + imiquimod) generated significantly higher anti-RBD antibody titers with a half maximal effective concentration (EC_50_) at a calculated mean dilution of 1:29,222 at Day 28, 3-fold higher compared with the RBD only vaccine at a calculated mean dilution of 1:10,924 (Fig. 1a). Moreover, sera were examined by live SARS-CoV-2 focus reduction neutralization test (FRNT) and surrogate virus neutralization tests (VNT) assay. In comparison to RBD sera, RBD+imiquimod sera produced significantly higher viral neutralization activities at day 28 in the live virus FRNT (*p* < 0.01) (Fig. 1c, Supplementary Fig. 2) and the surrogate VNT (*p* < 0.01) (Fig. 1d). Surprisingly, sera’s responses were above the majority and mean dilution of the EC_50_ and 50% inhibitory concentration (IC_50_) of convalescent COVID-19 patients, suggesting a possibly longer period of immune protection following vaccination (Fig. 1a, c, d).

**Fig. 1:**
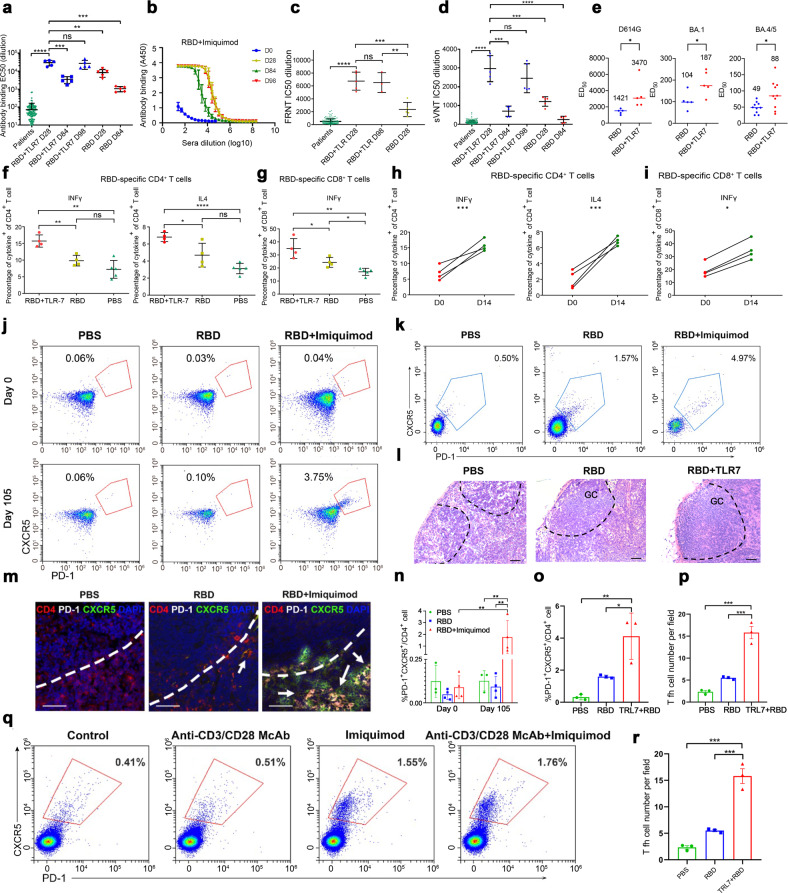
Enhanced anti-RBD Antibody and immune responses in RBD+imiquimod vaccine. **a** Comparison of EC_50_ values among sera samples from 192 convalescent COVD19 patients and two groups of monkeys: the RBD+imiquimod group at D28, D84, D98; the RBD only group at D28, D84. **b** A composite graph of anti-RBD antibody-binding values in NHPs’ sera immunized with RBD+ imiquimod vaccine at different time points of D0, D7, D14, and D84 and an ELISA test is done 2 weeks after immunization at different time points of D14, D28, and D98. *p < 0.05, **p < 0.01, ****p < 0.0001, “ns” denoted “none significant”, results were calculated by an unpaired Student’s *t*-test. **c** A scatter plot of an FRNT assay of live SARS-CoV-2 virus showing a comparison among IC50 values of sera samples from 192 convalescent COVD19 patients and NHPs immunized with the RBD+imiquimod at the Day 28 and 98 post-immunization or immunized with RBD only at the Day 28 post-immunization. **d** A scatter plot in an sVNT assay showing a comparison among IC50 values of sera samples from 192 COVID-19 patients and NHPs immunized with the RBD+imiquimod at Day 28, 84, and 98 post-immunization or immunized with RBD only at the Day 28 and 84 post-immunization. *p < 0.05, **p < 0.01, ****p < 0.0001, “ns” denoted “none significant”. **e** Neutralization activities of the immune sera against D614G, Omicron BA.1, and BA.4/5 variants. Neutralization activities were measured by pseudovirus assay, and the immune Sara was from NHPs vaccinated with an RBD protein vaccine (*n* = 5) or an RBD protein vaccine with imiquimod adjuvant (*n* = 5) against SARS-CoV-2 variants. **f**, **g** Cytokine fraction of different NHP groups at day 7 post-vaccination. **h**, **i** Comparison of cytokine fraction changes from before (D0) and after (D14) stimulation by an RBD antigen in lymphocytes from sera samples from RBD+ imiquimod vaccinated NHPs, *n* = 3. *p < 0.05, **p < 0.01, ****p < 0.0001, “ns” denoted “none significant”, results were calculated by an unpaired Student’s *t*-test. **j**, **n** Blood samples of NHP were collected at Day 0 and 105 post-immunization. PBMCs were isolated and detected by flow cytometry. CD4^+^ CXCR5^+^PD1^+^ staining was used to determine Tfh cells. The representative for the frequency of Tfh cells in CD4^+^ T cells from different vaccinated NHP groups at the Day 0 and 105 post-immunization (*n* = 4). Data were analyzed with a two-way ANOVA test and shown as the mean ± SEM (**p < 0.01). **k**, **o** The representative for the frequency of CD4^+^CXCR5^+^PD1^+^ T in the lymph nodes (LN) of different vaccinated NHP groups at Day 105 post-immunization (*n* = 3). Data were analyzed with a one-way ANOVA test and shown as the mean ± SEM (*p < 0.1, **p < 0.01). **l** Lymph node biopsies from different vaccinated NHP groups were collected on Day 105 post-immunization. Images for H&E staining of lymph node biopsies were captured at 200× magnification. Germinal center (GC) areas are highlighted by the dotted line (*n* = 3). Bar = 100 μm. **m**, **p** Representative merged images for the lymph node biopsies from different vaccinated NHP groups at Day 105 post-immunization. CD4^+^CXCR5^+^PD1^+^ Tfh in T–B border cells were marked with an arrow. Tfh cells in 5 fields of two sections for each separate animal were counted. Anti-CD4 (red), anti-CXCR5 (green), anti-PD-1 (white), and DAPI (blue) are presented (*n* = 3). Bar = 100 μm. Data were analyzed with a one-way ANOVA test and shown as the mean ± SEM (***p < 0.001). **q**, **r** The representatives for the dot of Tfh cells after 72 h stimulation of inguinal LN single cell suspension isolated from a normal cynomolgus. LN cells were divided into four parts, then cultured in the absence or presence of anti-CD3 and CD28 mAbs (2 and 2 μg/ml, respectively) with/without imiquimod. Data were analyzed with a one-way ANOVA test and shown as the mean ± SEM (**p < 0.01, ***p < 0.001, “ns” denoted “none significant”)

Dynamic changes in the virus-neutralizing activities were monitored over time. Though RBD+imiquimod enhanced the SARS-CoV-2 pseudovirus neutralizing activity, at 84 days post-vaccination, we found this activity was reduced by over 80% compared with day 28 (Fig. 1a). This reduction in antibody titer was also seen on enzyme-linked immunosorbent assay and surrogate VNT assay (Fig. 1b, d, Supplementary Fig. 1). However, a booster dose with imiquimod adjuvant administered at Day 84 rapidly restored neutralizing antibody titer, since the titer was as high as which at Day 28 when measured at 14 days post booster (Day 98) (Fig. 1a–d, Supplementary Fig. 1).

We next investigated the neutralization properties of RBD+ imiquimod vaccination against the D614G variant and newly emerged Omicron variants BA.1 and BA.4/5. Neutralizing activity of immunized sera against pseudovirus-carrying mutations of different variants was measured. Sera from the RBD+imiquimod group had an approximately two times higher neutralizing activity than that from the RBD group against D614G, Omicron BA.1, and BA.4/5 variants, though neutralizing titers of either vaccine was reduced dramatically (over 10 times) in the Omicron variants (Fig. 1e).

In addition to B-cell mediated antibody responses, cell-mediated immune responses involving CD4 and CD8 T cells are likely to play important roles in the clearance of SARS-CoV-2 infection and maintenance of protective immunity. Lymphocytes from NHPs were collected on day 0 prior to and day 7 post the first vaccination, and their cytokine responses, including IFN-γ and IL-4 production, were assayed. CD4^+^ or CD8^+^ T cell effectors were stimulated overnight with the same RBD antigen used in vaccination. All monkeys receiving prime-boost vaccination mounted RBD-specific T cell responses. Using flow cytometry, the number of intracellular CD4^+^IL4,^+^ CD4^+^IFN-γ, CD8^+^IFN-γ lymphocytes were increased in the RBD^+^imiquimod vaccinated group compared with those in the RBD-only vaccinated group (*p* < 0.05) and the sham (PBS) control group (*p* < 0.01) (Fig. 1f, g, Supplementary Figs. 3 and 4). We also compared changes in CD4^+^ and CD8^+^ T cell responses between day 0 prior to and day 14 post vaccination and observed significant increases in the number of CD4^+^IL4,^+^ CD4^+^IFN-γ, CD8^+^IFN-γ lymphocytes in the RBD+imiquimod vaccinated NHPs (Fig. 1h, i). These findings support the hypothesis that vaccination with RBD+imiquimod could effectively trigger cellular immune responses against the virus.

CD4^+^ helper T cells possess various effector functions, in which CD4^+^CXCR5^+^PD-1^+^ follicular T helper (Tfh) cells are a specialized subset that plays an important role in humoral immunity.^4^ We measured PBMC stained for CD4^+^CXCR5^+^PD-1^+^ Tfh cells at day 0 and day 105. There were negligible circulating Tfh cells at day 0 in all 3 groups. However, RBD+imiquimod immunized sera contained significantly more Tfh at day 105 compared to PBS-injected and RBD immunized sera (Fig. 1j, n, Supplementary Fig. 5a).

We then randomly selected 3 NHPs from each vaccination group to sacrifice at day 105 and collected their inguinal lymph nodes to investigate the fraction of Tfh cells using flow cytometry and immunofluorescence staining. The remaining two HNPs in each group were kept for long-term evaluation for any side effects. In agreement with the PBMC data, the frequency of CD4^+^CXCR5^+^PD-1^+^ Tfh cells in the inguinal lymph nodes was significantly higher in the RBD+ imiquimod group compared to those in the RBD alone group (*p* < 0.05) and the PBS group (*p* < 0.01) (Fig. 1k, o, Supplementary Fig. 5b). H&E staining of lymph node biopsies showed large germinal centers in RBD+ imiquimod-immunized group compared to small germinal centers the RBD group and the PBS control group (Fig. 1l). Immunofluorescent staining of PD-1 and CXCR5 also showed increased CD4^+^CXCR5^+^PD-1^+^ Tfh cells at the T-B border in LNs from the RBD+imiquimod group (Fig. 1m, p).

To further evaluate the effect of TLR7 agonist on Tfh cells, cells from the inguinal lymph nodes from unimmunized Cynomolgus macaques were isolated and stimulated with or without imiquimod in the absence or presence of anti-CD3/anti-CD28 monoclonal antibodies (mAb). As shown in Fig. 1q, supplementary Fig. 5c, r, stimulation with anti-CD3/CD28 mAb did not significantly enhance Tfh cells. Imiquimod significantly raised Tfh cell proportion irrespective of anti-CD3/CD28 stimulation. These results demonstrate that the TLR7 agonist imiquimod may act via the enhancement of Tfh-mediated immunity.

Altogether, these findings indicate that the RBD vaccine with a TRL7 adjuvant induced high levels of neutralizing antibodies, as well as enhancement of functional cellular immune responses involving CD4 and CD8 T cells. Given the short half-life and minimal systemic absorption of imiquimod, we believe that imiquimod activates TRL7 on the site of application to trigger enhanced vaccine-induced immunity.

## Supplementary information

Supplemental Material - clean version

## Data Availability

The data/materials used in the current study are available from the corresponding authors upon reasonable request.
