# Supplementary material for: Enhancement of SARS-CoV-2 vaccine-induced immunity by a Toll-like receptor 7 agonist adjuvant
Source: Signal Transduct Target Ther. 2023 May 24;8:213. doi: 10.1038/s41392-023-01485-6 (PMC10206358; doi:10.1038/s41392-023-01485-6)
Supplement: Supplementary file 1 — Supplemental Material - clean version [file 41392_2023_1485_MOESM1_ESM.docx]

Supplementary Materials for

Enhancement of SARS-CoV-2 vaccine-induced immunity by a Toll-like receptor 7 agonist adjuvant

Gen Li, Meixing Yu, Qiong Ke, Jing Sun, Yanwen Peng, Chuanfeng Xiong, Olivia Monteiro, COVID-19 Infection and Immunity Investigation Group, Jincun Zhao, and Andy P. Xiang

Correspondence to: [superleegen@hotmail.com](mailto:superleegen@hotmail.com)

**This PDF file includes:**

Materials and Methods

Figures. S1 to S5

**Materials and Methods**

Vaccine and booster procedure in NHPs

All procedures involved in the non-human primates study were reviewed and approved by the Institutional Animal Care and Use Committee of Institute of Sun Yat-sen University. Fifteen adult non-human primates Cynomolgus macaques (*Macaca fascicularis*) (5-9 years old) were used for the vaccine study and assigned into the following groups: (a) the group immunized with 20 μg RBD protein with Al(OH)_3_ per dose (n=5); (b) the group immunized with 20 μg RBD protein with Al(OH)_3_ plus topical imiquimod (n=5); (c) with PBS sham injection (control treatment, n=5). Non-human primates were given a topical application of imiquimod cream as 5% Aldara cream (100 mg) followed immediately by immunization with an intradermal injection on day 0, day 7, day 14. Sera samples were collected at D7, D14, D28, D84 and D98 for an analysis. A booster injection with 20 μg RBD protein with Al(OH)_3_ was given to the RBD+ Imiquimod treated group at day 84. The detail methods for vaccination, booster administration and NHP experiments can be found in our recent report ^1^.

Pseudovirus construction Plasmids

The psPAX2 was purchased from Addgene, and the pLOVE-Luciferase-EGFP was from GenScript (Nanjing, China); the full-length Spike gene (S) from the SARS-CoV-2 (previously 2019-nCoV) strain Wuhan-Hu-1 (GenBank: MN908947) was codon-optimized, synthesized, and cloned into pCAGGS vector (pCAGGS S(1-1254aa)) with seamless cloning by GenScript. A Site-directed mutagenesis approach was used to generate WT D614, D614G, and Omicron BA.1, BA.4/5 mutants, based on a high-fidelity DNA polymerase Mix (P525, Vazyme), with the condition as follows: 95℃ 5min, 95℃ 30s, 56℃ 30s, 72℃ 4min for 28 cycles, 72℃ 10min. Subsequently, we purified the exact size of Spike gene PCR product by gel extraction, and then used Exnase II (C214, Vazyme) to circularize the linearized products. Then, the designed circularized mutant plasmids were transformed into DH5α competent cells, and single clones were select to cultivate recombinant plasmids in culture.

Cell culture

HEK293T cells and ACE2-293T cells (cells transfected with human ACE2) were purchased from ProCell (Wuhan, China); 293T cells were maintained in Dulbecco’s Modified Eagle Medium (Invitrogen) supplemented with 10% fetal bovine serum (FBS; Gibco, Rockville, MD, USA), at 37 °C in 5% CO2 at a concentration of100 mg/mL of streptomycin, and 100 unit/mL of penicillin. Under the same conditions, HEK293T cells transfected with human ACE2 (293T-ACE2) were cultured with the addition of G418 (0.5 mg/mL) to the medium.

Production and titration of SARS-CoV-2 S pseudoviruses

We generated either wild type SARS-CoV-2 S, S-D614G, Omicron BA.1 and BA.4/5variant pseudotyped virus with a luciferase reporter via an HIV-1 backbone as described previously^2^. Specifically, 5x10^6^ HEK293T cells in 100mm dish were co-transfected with 12ug pLOVE-luciferase-EGFP plasmid, 6ug psPAX2 and 2ug recombinant SARS-CoV-2 S plasmids or SARS-CoV-2 S-D614G plasmid. Transfection was carried out with the lipofectamine 3000 transfection reagent (Invitrogen) in accordance with the manufacturer’s instructions. (A: 1ml OPTI-DMEM + 40ul lipofectamine 3000; B: 1ml OPTI-DMEM + 40ul P3000 + Plasmids; Mix A and B, incubate at R.T. for15min, and then add the Mix to the culture dish). The medium for transfected cells were replaced by a fresh Medium (10ml) after ~8 h. The supernatant containing SARS-CoV-2 pseudoviruses was harvested at 48h and 72h after the initial transfection and filtered through a 0.45um filter. Subsequently, pseudoviruses were concentrated by a centrifugal ultrafiltration device, and then the viruses for one package were dissolved with 50ul medium. pseudoviruses were titrated by qRT-PCR with TransLvTM Lentivirus qPCR Titration Kit (FV201, Transgen).

Psuedovirus neutralization assay

Neutralization measurement was performed through lentivirus particles pseudotyped with full-length SARS-CoV-2 S containing either wild type, D614G, Omicron BA.1, and BA.4/5 spike protein variants as described previously^2^. As this was the only difference between the pseudoviruses, any change in phenotype was directly attributed to mutations. Assays were performed with nearly the same amounts of input virus doses (relative light units (RLU) in virus control wells were 501-840 x background in all assays).

For each neutralization assay, 50ul of medium containing pseudoviruses (~1.2×10^5^ vg) was incubated using media or the serially diluted sera derived from HNPs that were vaccinated by RBD or RBD+imiquimod, and convalescent patients at 37℃ for 1h, before being added to the 96-well plates containing ACE2-293T cells. After 12h of infection, fresh culture medium was added to each well. Luciferase activity was measured 48h after the infection with ONE-GloTM Luciferase Assay System (E6120, Promega). Recruiting and acquiring sera from SARS-CoV-2 convalescent patients and using these sera in this study was approved by the University Hospital Clinical Research Ethics Committee of the Macau University of Science and Technology (Approval reference: UH/CREC/2022/01).

Live SARS-CoV-2 focus reduction neutralization test and calculation of IC50

SARS-CoV-2 focus reduction neutralization test (FRNT) was performed in a certified biosafety level 3 lab as previously described ^3,4^. Fifty μl ten-fold dilution of serum samples were serially (1:4) diluted, mixed with 50 μl of SARS-CoV-2 (150-200 focus forming unit, FFU) in 96-well microwell plates and incubated for 1 hour at 37˚C. Mixtures were then transferred to 96-well plates seeded with Vero E6 cells (ATCC, Manassas, VA) for 1 hour at 37˚C. Inoculums were then removed before adding the overlay media (100 μl MEM containing 1.6% Carboxymethylcellulose, CMC). The plates were then incubated at 37˚C for 24 hours. Cells were fixed with 4% paraformaldehyde solution for 30 min, and permeabilized with 0.2% Triton X-100 and incubated with rabbit anti-SARS-CoV-N polyclonal IgG which was cross-reactive with SARS-CoV-2 (Cat: 40143-T62, Sino Biological, Inc, Beijing) for 1 hour at 37˚C. and incubated with HRP-labelled goat anti-rabbit secondary antibody (Code: 111-035-144, Jackson ImmunoResearch, West Grove, PA) for 1 hour at 37˚C. The foci were visualized by KPL TrueBlue Peroxidase substrates (Seracare Life Sciences Inc, Milford, MA), and counted with an ELISPOT reader (CTL S6 Ultra, Cellular Technology Ltd, Shaker Heights, OH). FRNT_50_ was calculated using the 4-parameter logistic model.

Surrogate VNT assay

Anti-SARS-CoV-2 antibody titer was examined by using SARS-CoV-2 Surrogate Virus Neutralization Test kit (GenScript, L00847-A). In brief, the samples and controls are pre-incubated with horseradish peroxidase-receptor binding domain (HRP-RBD) at 37℃ for 30 min, which allowed the binding of SARS-CoV-2 neutralizing antibodies to HRP-RBD. Then the mixture was added to a capture plate that was pre-coated with the recombinant hACE2 protein, and incubated at 37℃ for 15 min. Only HRP-RBD that was not bound to the neutralizing antibody would be captured by hACE2 protein, otherwise it would be removed by washing. After washing steps, the TMB solution was added the plate was incubated in dark at room temperature. After 15 minutes, Stop Solution was added to quench the reaction. The final solution was read in dual wavelength microplate reader (Thermo, MULTISKAN GO) at 450 nm. The absorbance was inversely proportional to the titer of the anti-SARS-CoV-2 neutralizing antibody in the sample.

Flow cytometry

T cells was evaluated with flow cytometry as previously described ^5^. Lymphocytes of the 15 samples were isolated, because two of them were found to be clumped together (one from the RBD group and the other from the RBD+imiquimod group), the remaining 4 samples from RBD group, 4 samples from the RBD +imiquimod agonist group and 5 negative PBS controls were retained for the following procedure. The lymphocytes were cultured in RPMI medium 1640 supplied with 10％ (vol/vol) FBS, 100 U/ml penicillin, 100 μg/ml streptomycin for 16 h. Meanwhile, 5μg/ml S protein RBD was added to activate cells. 16 h later, 100ng/ml PMA (Sigma-Aldrich), 1ug/ml Ionomycin (Merk Millipore) and Brefeldin A (BD Biosciences) were administrated and incubated with lymphocytes for 4 hours. Cells were then washed in PBS (Gibco) and incubated with anti-CD8 and anti-CD4 for surface staining and with anti-IFNγ and anti-IL-4 for intracellular staining. All antibodies used in this experiment were purchased from BD Biosciences. Flow cytometry data were acquired on a BD FACSCanto system and analyzed using the FlowJo V10 software.

Blood samples of NHP were collected on the day of vaccination, at 105d after the first vaccination, blood samples and inguinal lymph nodes were collected. Peripheral blood mononuclear cells (PBMCs) were isolated by density centrifugation, single cell suspension of LNs were obtained by meshing through 100µm cell strainer (BD; San Jose, USA). Cells were stained with anti-CD4-PE(clone A161A1), anti-CXCR5-FIT(clone RF8B2) and anti-PD-1-APC(clone MIH4), then detected on The Beckman Coulter Life Sciences CytoFLEX benchtop flow cytometer, and analyzed by FlowJo V10 software. CD4^+^ CXCR5^+^PD1^+^ staining was used to determine Tfh cells. Above antibodies were purchased from Biolegend.

Histochemical staining and Immunofluorescence analysis of lymph node biopsy tissues

After 105d from the first vaccination, inguinal lymph nodes were gathered and fixed in 4% formalin, sectioned to 10 μm thickness. Some sections were stained with hematoxylin and eosin (H&E), the other sections were incubated with anti-CD4 (clone A161A1 ), anti-CXCR5（clone RF8B2）and anti-PD-1（clone MIH4）antibodies. All antibodies were purchased from Biolegend. Imaging was captured with a Zeiss LSM 880 confocal microscope (Zeiss，Germany) at 200x magnification. Adobe Photoshop software (Version 7.0; Adobe Systems) was used to process and merge the images. Cells in the T-B cell border was determined by immunofluorescence antibodies, specifically, anti-CD4 (red), anti-PD-1 (white), and anti-CXCR5 (green), respectively. CD4^+^CXCR5^+^PD1^+^ Tfh cells in 5 fields of two section for each separate animal were counted. The colocalization of CD4, PD-1, CXCR5 is evaluated by the overlay color light orange (red+green+white) in the merged images analyzed by Bitplane Imaris v7.4.2.

Measurement of total binding IgG and IgM against SARS-CoV-2

The total binding IgG and IgM antibodies against SARS-CoV-2 were measured using a commercially available Magnetic Chemiluminescence Enzyme Immunoassay kit as previous reported ^6^ (Bioscience, Chongqing, China) according to the manufacturer's instructions. Antibody levels were expressed as the ratio of the chemiluminescence signal over the cutoff value (S/CO). A S/CO value of >1.0 for either IgG or IgM was regarded as positive.

In vitro Tfh Stimulation experiment

Inguinal lymph nodes from normal NHP were obtained and grinded by gentle mechanical disruption, the cells meshed through 100µm cell strainer (BD; San Jose, USA) to obtain single cell suspension. After lysing red blood cells, the cells were washed with RPMI-1640 complete medium and divided into four parts. The first one was culture alone, the second one added anti-human CD3 monoclonal antibody and anti-human CD28 monoclonal antibody, the third one added Imiquimod, the last one added anti-CD3 and anti- CD28 monoclonal antibodies combined with Imiquimod. After 72h culture, cells were gathered and stained by anti-CD4-PE, anti-CXCR5-FITC and anti-PD-1-APC. The stained cells were detected on The Beckman Coulter Life Sciences CytoFLEX benchtop flow cytometer and analyzed by FlowJo V10 software.

**Figure S1.**

**Figure S1. a**. Anti-RBD Antibody binding values in NHPs’ sera immunized with an RBD + imiquimod vaccine at different time points (D0, D14, D28, D84, D98) in individual NHPs, n=3. **b**. Anti-RBD Antibody binding values in NHPs’ sera samples immunized with RBD only vaccine at different time points (D0, D14, D28, D84) in individual NHPs, n=3.

**Figure S2.**

**Figure S2.** Picture showing a 96 well microtiter plate in an FRNT assay. Each blue spot represents a positive viral infection event. See the Method for details.

**Figure S3.**

**Figure S3. a.** Picture of flow cytometry showing IL4 and INFγ fraction values of CD4^+^ T cells from different vaccinated NHP groups cultured with RPMI1640 and an RBD antigen, and stimulated by PMA. **b.** Gating strategy

**Figure S4.**

**Figure S4. a**. Picture of flow cytometry showing INFγ fraction values of CD8^+^ T cells from different vaccinated NHP groups cultured with RPMI1640 and an RBD antigen, and stimulated by PMA. **b**. Gating strategy.

**Figure S5.**


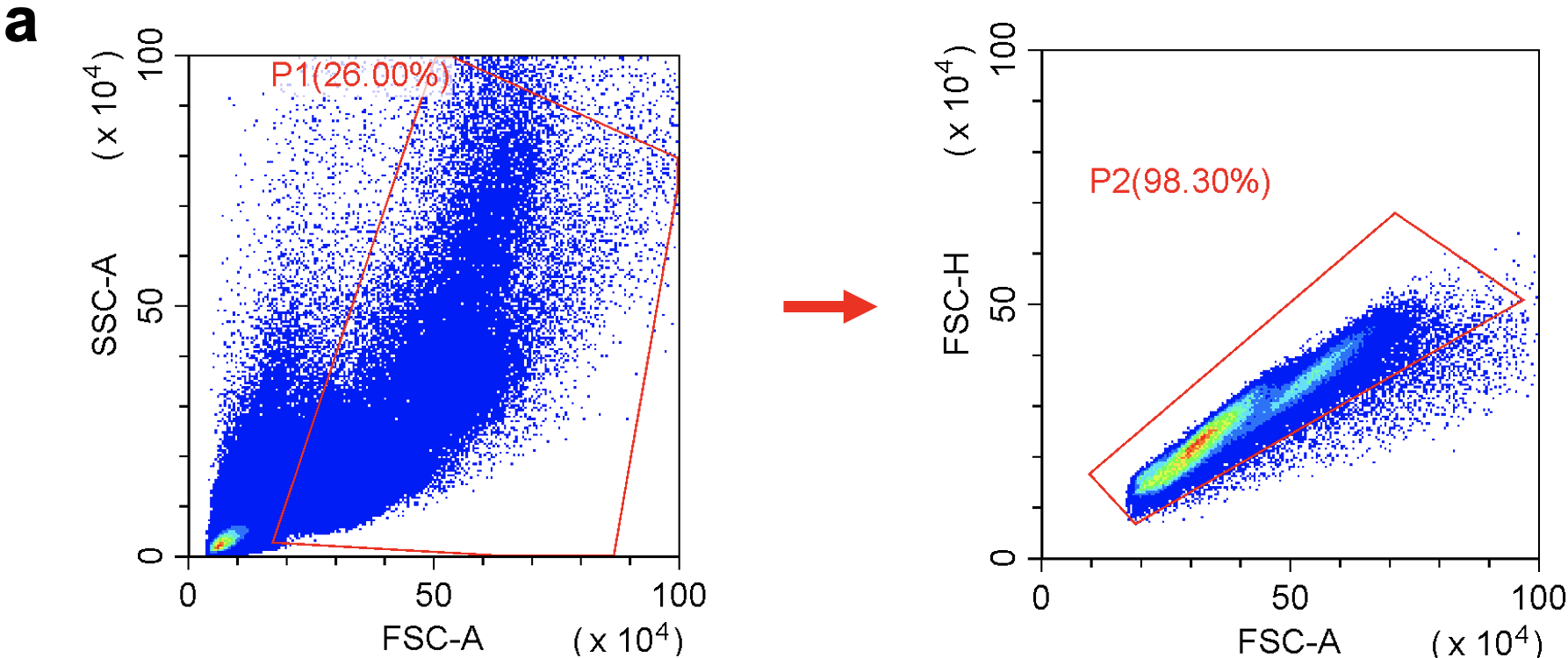


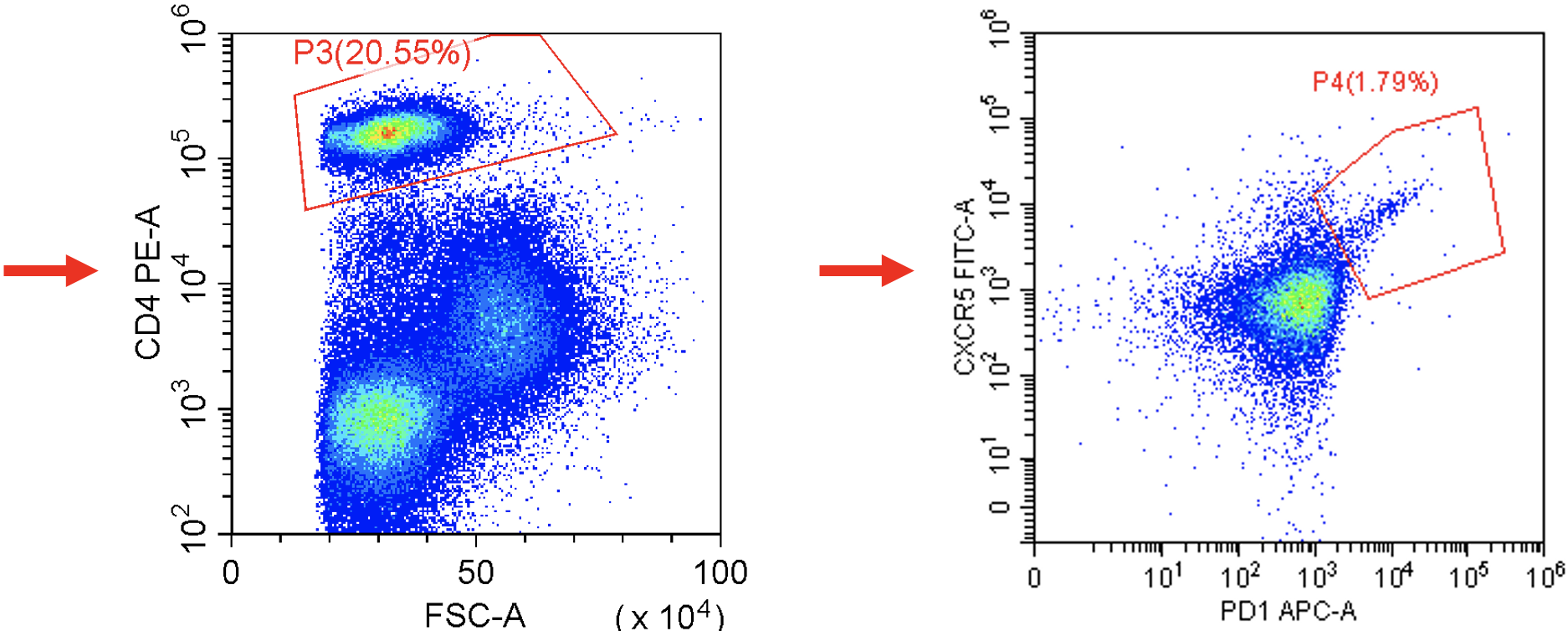


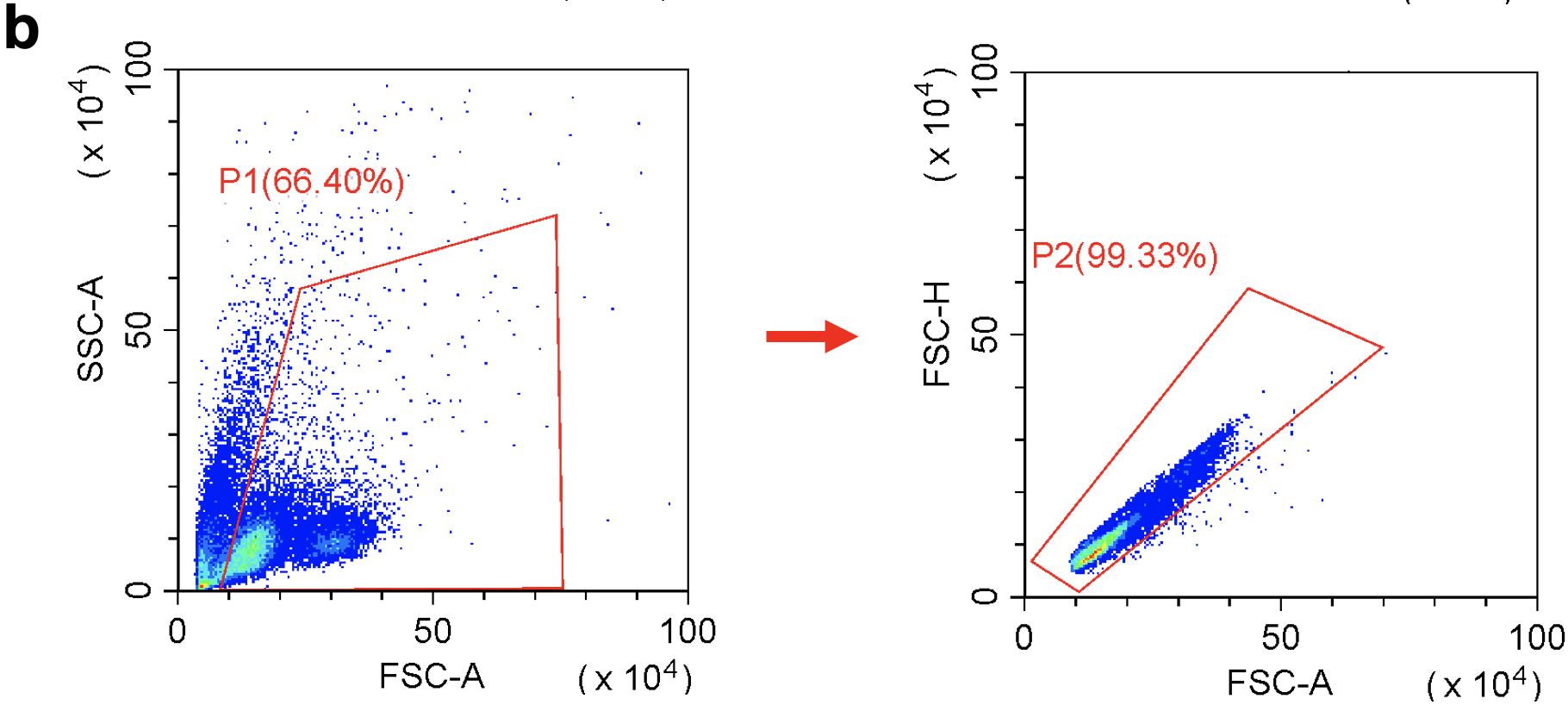

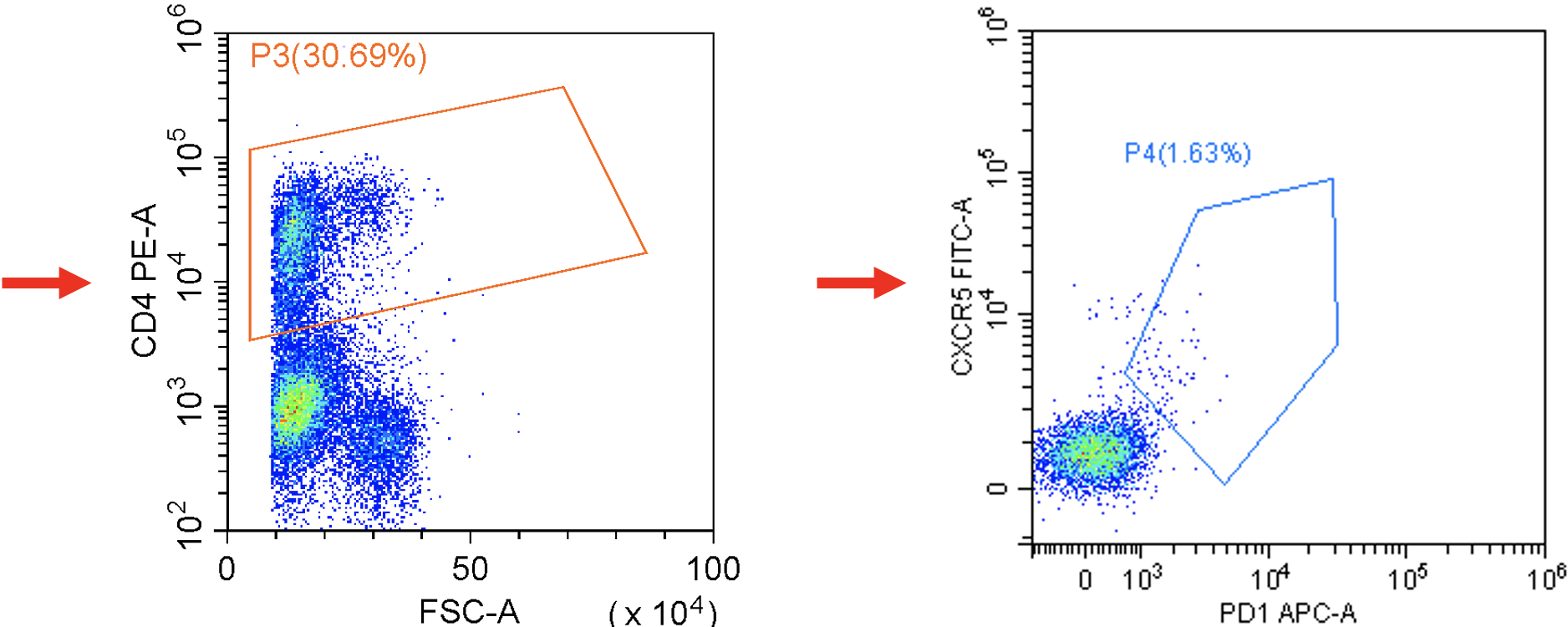


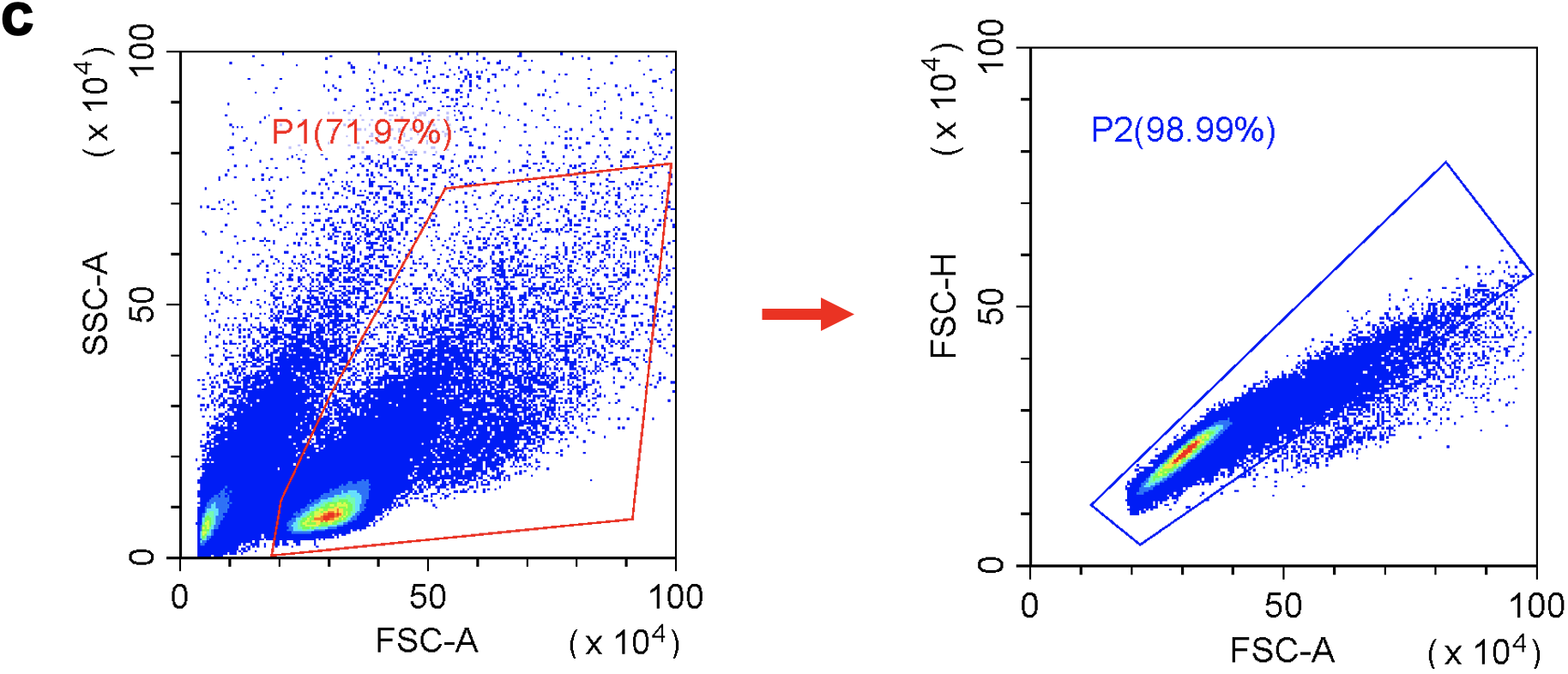


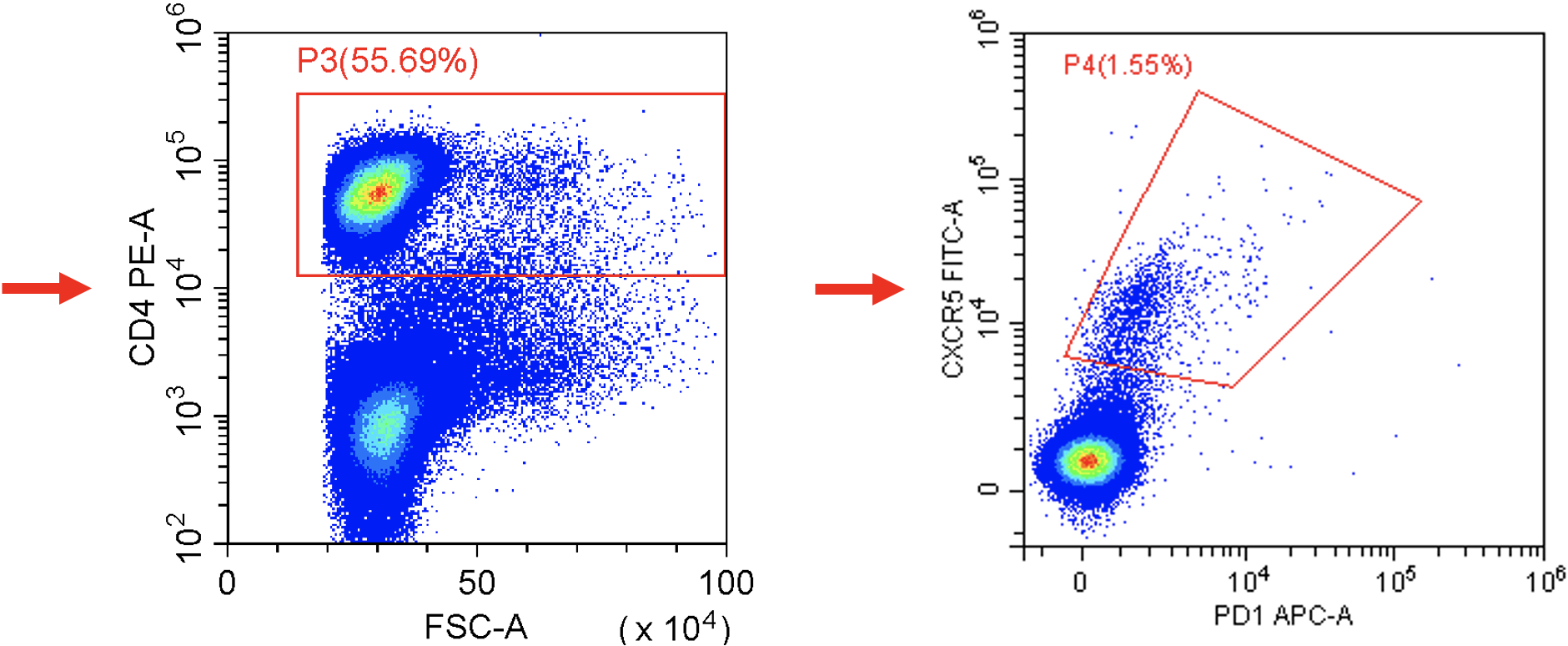


**Figure S5. a**. Gating strategy for Figure 1j. **b**. Gating strategy for Figure 1k. **c**. Gating strategy for Figure 1q.

**References**

1 Sahin, U. *et al.* COVID-19 vaccine BNT162b1 elicits human antibody and TH1 T cell responses. *Nature*. **586**, 594-599, (2020).

2 Corbett, K. S. *et al.* SARS-CoV-2 mRNA vaccine design enabled by prototype pathogen preparedness. *Nature*. **586**, 567-571, (2020).

3 Wang, Y. *et al.* Kinetics of viral load and antibody response in relation to COVID-19 severity. *J Clin Invest*. **130**, 5235-5244, (2020).

4 Sun, J. *et al.* Generation of a Broadly Useful Model for COVID-19 Pathogenesis, Vaccination, and Treatment. *Cell*. **182**, 734-743.e735, (2020).

5 Xiao, Y. *et al.* Adsorption of recombinant poxvirus L1-protein to aluminum hydroxide/CpG vaccine adjuvants enhances immune responses and protection of mice from vaccinia virus challenge. *Vaccine*. **31**, 319-326, (2013).

6 Xu, X. *et al.* Seroprevalence of immunoglobulin M and G antibodies against SARS-CoV-2 in China. *Nat Med*. **26**, 1193-1195, (2020).
